# A screen of pharmacologically active compounds to identify modulators of the Adgrg6/Gpr126 signalling pathway in zebrafish embryos

**DOI:** 10.1111/bcpt.13923

**Published:** 2023-07-16

**Authors:** Anzar Asad, Nahal O. Shahidan, Antonio de la Vega de León, Giselle R. Wiggin, Tanya T. Whitfield, Sarah Baxendale

**Affiliations:** ^1^ School of Biosciences University of Sheffield Sheffield UK; ^2^ Information School University of Sheffield Sheffield UK; ^3^ Sosei Heptares, Steinmetz Building, Granta Park Cambridge UK; ^4^ Sheffield Zebrafish Screening Facility, School of Biosciences University of Sheffield Sheffield UK

**Keywords:** Adgrg6, chemical screen, Gpr126, inner ear, LOPAC library, myelination, zebrafish

## Abstract

Adhesion G protein‐coupled receptors (GPCRs) are an underrepresented class of GPCRs in drug discovery. We previously developed an in vivo drug screening pipeline to identify compounds with agonist activity for Adgrg6 (Gpr126), an adhesion GPCR required for myelination of the peripheral nervous system in vertebrates. The screening assay tests for rescue of an ear defect found in *adgrg6* ^*tb233c−/−*^ hypomorphic homozygous mutant zebrafish, using the expression of *versican b* (*vcanb*) mRNA as an easily identifiable phenotype. In the current study, we used the same assay to screen a commercially available library of 1280 diverse bioactive compounds (Sigma LOPAC). Comparison with published hits from two partially overlapping compound collections (Spectrum, Tocris) confirms that the screening assay is robust and reproducible. Using a modified counter screen for *myelin basic protein* (*mbp*) gene expression, we have identified 17 LOPAC compounds that can rescue both inner ear and myelination defects in *adgrg6* ^*tb233c−/−*^ hypomorphic mutants, three of which (ebastine, S‐methylisothiourea hemisulfate, and thapsigargin) are new hits. A further 25 LOPAC hit compounds were effective at rescuing the otic *vcanb* expression but not *mbp*. Together, these and previously identified hits provide a wealth of starting material for the development of novel and specific pharmacological modulators of Adgrg6 receptor activity.

## 1 INTRODUCTION

Adgrg6/Gpr126 is a conserved developmental modulator of myelination of the vertebrate peripheral nervous system (PNS).^1^, ^2^, ^3^ Homozygous loss‐of‐function of this adhesion class G protein‐coupled receptor (aGPCR) in zebrafish or mouse reduces Schwann cell expression of myelin basic protein, a major component of the myelin sheath.^3^, ^4^, ^5^, ^6^ Human mutations of *ADGRG6* are also associated with hypomyelination of the PNS and are causative for a lethal contracture syndrome, arthrogryposis multiplex congenita (AMC),^2^, ^7^ while other studies have linked variants in *ADGRG6* to a wide range of diseases, including adolescent idiopathic scoliosis and cancer (reviewed in Baxendale et al.^8^). In the zebrafish, Adgrg6 also has important roles in morphogenesis of the developing ear.^9^, ^10^ Zebrafish homozygous *adgrg6* ^*−/−*^ mutants fail to form semicircular canal ducts and exhibit persistent otic expression of various extracellular matrix genes, including core protein genes for the proteoglycan Versican, normally transiently expressed in the developing ears of wild‐type embryos.^10^

Recent work has provided insight into the structure, ligands and mechanism of action of Adgrg6.^11^, ^12^, ^13^, ^14^, ^15^, ^16^, ^17^ A chemical ligand that binds directly with Adgrg6 could provide a valuable tool to enable isolation of the receptor in its in vivo conformation, paving the way for further structural and mechanistic insights, as well as having potential for therapeutic use. Modulators of Adgrg6 pathway activity could also provide useful tools or therapeutics to modulate myelination following peripheral nerve injury or in *ADGRG6*‐linked disease conditions.^18^, ^19^, ^20^, ^21^ Zebrafish are a valuable whole‐organism model in which to screen for such compounds.^21^, ^22^, ^23^, ^24^, ^25^ Drug screening in the zebrafish takes into consideration absorption, circulation, metabolism and toxicity of compounds during the early stages of the drug discovery pipeline, properties that can often prove to be a challenge when advancing to rodent studies following validation of hits in cell culture (reviewed in Baxendale et al.^26^ and Patton et al.^27^).

**FIGURE 1:**
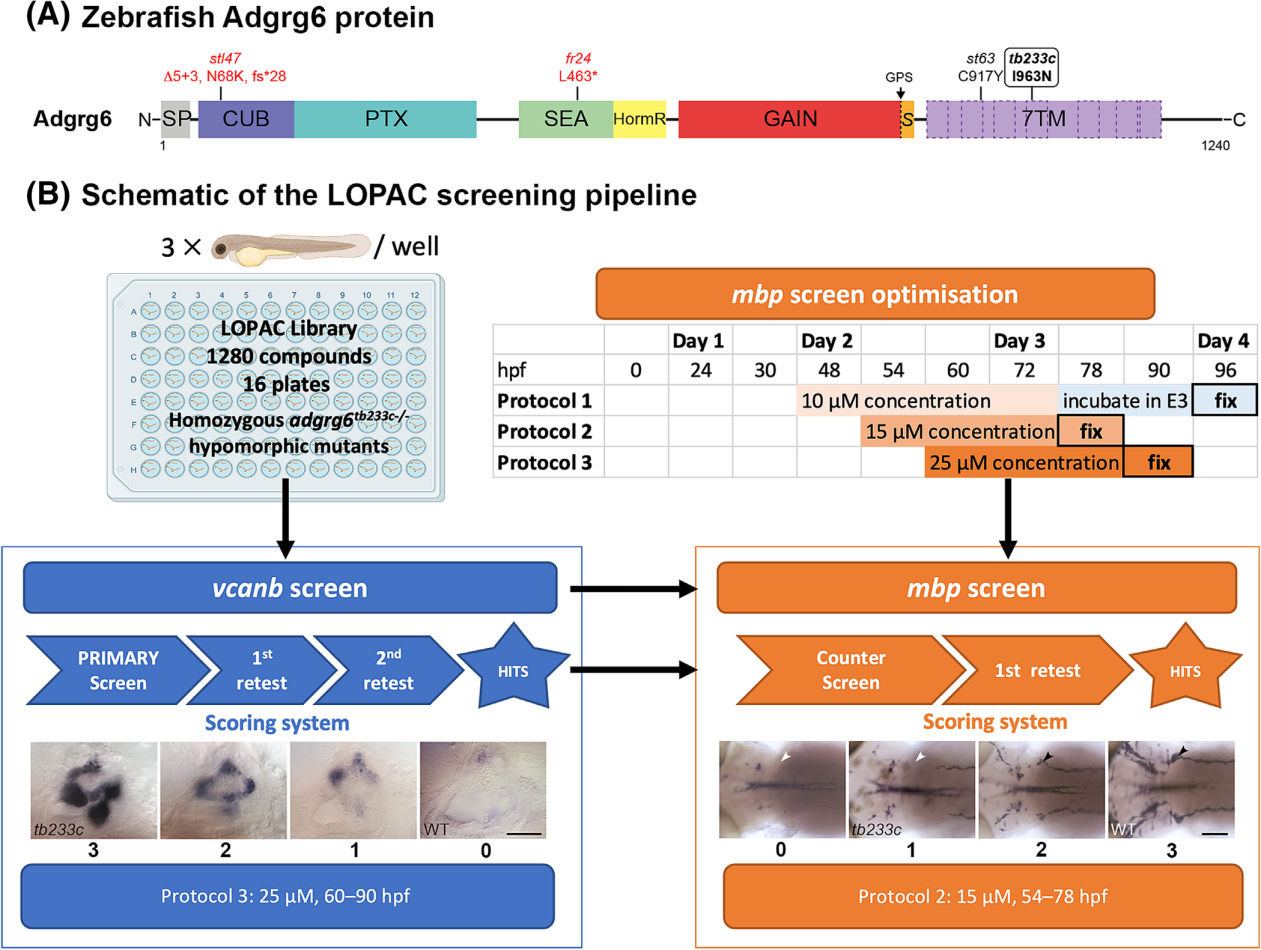
Schematic of zebrafish Adgrg6 alleles and the LOPAC screening pipeline. (A) Schematic of the Adgrg6 protein and the following domains: Signal peptide (SP), complement C1r/C1s, Uegf, BMP1 (CUB), pentraxin (PTX), sperm protein, enterokinase, and agrin (SEA), hormone receptor (HormR), GPCR autoproteolysis‐inducing (GAIN), Stachel sequence (S), and 7‐pass transmembrane (7TM) (for details, see references in.^8^ The GPCR autoproteolytic site (GPS) is shown (black arrow). The position of the amino acid change in the *tb233c* allele is shown,^10^ together with other alleles^3^, ^10^, ^16^ discussed in the text. Hypomorphic missense alleles and the amino acid changes are shown above in black; truncating nonsense mutations are shown in red. (B) Schematic of the screening pipeline. The LOPAC library was screened on homozygous *adgrg6* ^*tb233c−/−*^ hypomorphic mutant zebrafish embryos. Initially, all compounds were tested using the primary *vcanb* screen (blue) followed by two retests. Confirmed hits and some additional selected compounds (see text) were then tested in an optimized counter screen for *mbp* expression (orange). Images show the expression of *vcanb* in the developing inner ear (lateral views with anterior to the left and dorsal to the top, taken from Diamantopoulou et al.^23^) and *mbp* expression in the posterior lateral line ganglia (arrowheads) and nerves (dorsal views of the embryo; anterior to the left). Scores shown below the images were assigned as described previously.^23^ Scale bars: *vcanb* images, 50 μm; *mbp* images, 100 μm.

In a previous study, we utilized the otic expression of *versican b* (*vcanb*) as a screening assay to identify compounds from the Tocris Total and Spectrum libraries that could rescue (down‐regulate) expression in *adgrg6* ^*tb233c−/−*^ hypomorphic missense mutants (Figure 1A). A subgroup of hit compounds could also partially restore (up‐regulate) *myelin basic protein* (*mbp*) mRNA expression in peripheral nerves of mutant embryos, building confidence in the compounds as candidate agonists of Adgrg6.^23^ An independent screen of the Pharmakon library—for rescue of *mbp*‐driven GFP fluorescence in peripheral nerves of *adgrg6* ^*st63−/−*^ hypomorphic missense mutants—identified additional hit compounds.^22^ Both studies used more severe loss‐of‐function *adgrg6* alleles, with predicted early protein truncations, to differentiate activity of hit compounds^22^, ^23^ (Figure 1A). For example, molecules of the gedunin class, which includes known agonists of other aGPCRs,^28^ were effective in rescuing otic *vcanb* expression in a hypomorphic missense allele (*adgrg6* ^*tb233c−/−*^), but not in a protein‐truncating allele (*adgrg6* ^*fr24−/−*^).^23^ These findings make gedunins promising candidates for compounds that bind directly to the receptor.

In the current study, we have screened the structurally diverse Library of Pharmacologically Active Compounds (LOPAC; Sigma). This collection of 1280 drug‐like small molecules contains 603 compounds that are also represented in either or both of the Tocris and Spectrum libraries, and 677 unique compounds, of which 275 are structurally diverse from any compounds we have previously tested on *adgrg6* ^*−/−*^ mutant embryos. Our aims were twofold: To test compounds overlapping with the previously screened Tocris and Spectrum libraries to determine the reproducibility of their performance in our screening assay, and to screen the unique LOPAC compounds to identify novel modulators of the Adgrg6 signalling pathway.

## 2 RESULTS

### 2.1 Screen of the LOPAC library for compounds that mediate down‐regulation of otic *vcanb* expression in *adgrg6* hypomorphic mutants

A previous screen of two commercial small molecule libraries (Tocris Total, Spectrum) identified clusters of structurally related compounds that could rescue both the inner ear and the myelination defects in *adgrg6* ^*tb233c−/−*^ hypomorphic mutant embryos.^23^ We decided to extend the study to screen the Sigma LOPAC 1280 Library of Pharmacologically Active Compounds, which contains a diverse range of scaffolds and covers all major drug target classes. The LOPAC collection includes 603 compounds that overlap with the Tocris Total and Spectrum libraries, allowing us to test the reproducibility of compound performance in the assays used. The remaining 677 compounds, including 275 structurally novel compounds, have not previously been screened for rescue of the *adgrg6* ^*tb233c−/−*^ mutant phenotype.

**FIGURE 2:**
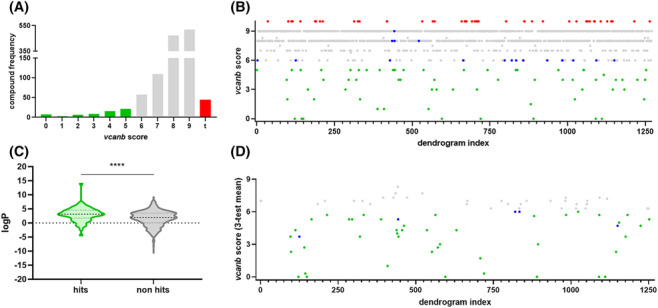
A primary drug screen of the LOPAC^1280^ library reveals 48 hit compounds that consistently down‐regulate otic *vcanb* expression in *adgrg6* ^*tb233c−/−*^ embryos. (A) Overview of *vcanb* staining scores in the primary screening assay. Green, hits (score <6); grey, non‐hits (score 6–9); red, toxic/corrosive compounds. (B) Total *vcanb* staining scores (summed from 3 embryos) from all compounds in the primary screen, including 64 initial hits (green). Library compounds are ordered along the *x* axis based on structural similarity. Red data points, toxic/corrosive compounds; grey, no effect on *vcanb* expression (non‐hits); green, reduced *vcanb* expression (initial hits); blue, additional compounds selected for retest based on structural similarity with hits. (C) Comparison of lipophilicity of initial hit (green) and non‐hit (grey) primary screen compounds. ****p < 0.0001, Welch's two‐tailed *t* test. (D) Retesting 82 compounds revealed 48 (green and blue) that consistently down‐regulated *vcanb* expression after two retests.

The LOPAC screen was performed by administering compounds at 25 μM to live *adgrg6* ^*tb233c−/−*^ hypomorphic mutant embryos (three per well) between 60 and 90 h post fertilization (hpf), followed by semi‐automated in situ hybridization to determine levels of otic *vcanb* expression (Figure 1, Protocol 3). This protocol matched the conditions of our screen of the Tocris Total and Spectrum libraries,^23^ ensuring comparability of results with those of the previous study. Otic *vcanb* expression was scored for each embryo as previously^23^ (Figure 1; hit score 0–2, non‐hit score 3). Compounds that gave a score of <6 (summed from all three embryos in a well) were recognized as initial hits, representing 5% (64/1280) of the compounds in the LOPAC library (Figures 2 and S1). Note that these initial hits could include false positives (e.g., compounds that down‐regulate gene transcription generically), which were eliminated later in a counter screen for *mbp* expression (see below); 4% (45/1280) of compounds were toxic or corrosive, whereas the majority (91%; 1161/1280) had no significant rescuing or other effect on *adgrg6* ^*tb233c−/−*^ hypomorphic mutant embryos at the administered concentration.

### 2.2 Selection of hit compounds for retests and further analysis

In addition to the 64 compounds identified as hits in the LOPAC primary screen, other approaches were used to select additional compounds of interest. Compounds were clustered based on structural similarity alongside the Tocris Total and Spectrum library compounds (Figure S1 and interactive network; see Section 4). This clustering revealed compounds that, although scored as non‐hits in the primary *vcanb* screening assay, were structurally related to identified hits. Those that scored on the margins of the hit threshold were selected for a retest. Overall, 18 additional compounds of interest were selected, all of which formed at least one structure‐based connection with an identified hit from across all three libraries, and returned a *vcanb* score of 6–8 in the primary screen.

In total, 82 compounds were retested in duplicate to identify compounds that reproducibly mediated down‐regulation of otic *vcanb* expression in *adgrg6* ^*tb233c−/−*^ hypomorphic mutant embryos (Figure 2B,D). After the retests, the hit threshold score was adjusted to include compounds consistently scoring ≤6 from a three‐test average (nine embryos). Of the initial hits, 67% (43/64) tested positive in the retests. By comparison, only 28% (5/18) of the additional compounds selected for retests reached the new threshold score. There was a clear positive correlation (*R* ^2^ = 0.7) between the two retest scores for each compound, instilling confidence in the reproducibility of the assay output (Figure S2). As expected, the data indicated that low‐scoring compounds from the primary screen (those that rescued the phenotype well) were more likely to be identified as hit compounds across all retests. Log *P* analysis of the LOPAC library revealed that hit compounds are more lipophilic than non‐hit compounds (Figure 2C).

### 2.3 An *mbp* counter screen reveals potential Adgrg6 pathway modulators

We tested for rescue of *mbp* expression levels in *adgrg6* ^*tb233c−/−*^ hypomorphic mutants as a counter screen to verify hits from the primary screen and to identify and eliminate any false‐positives. We first set out to optimize the assay conditions for rescue of *mbp* expression in *adgrg6* ^*tb233c−/−*^ mutant embryos. We trialled multiple treatment windows and compound concentrations, using two dihydropyridine hits (cilnidipine, nilvadipine) from our previous screen,^23^ together with IBMX (the primary screen positive control), as test compounds (Figure S3). We quantified *mbp* expression in Schwann cells around the posterior lateral line ganglion (PLLg), as expression in this location is reliably missing in *adgrg6* ^*tb233c−/−*^ mutant embryos.^10^, ^23^ A treatment window of 54–78 hpf, with a test compound concentration of 15 μM, was established as optimal (Figure 1, Protocol 2) and selected for the secondary screening assay. For rescue of the *tb233c* hypomorphic allele, this regime compared favourably with our previously published conditions (60–90 hpf, 25 μM)^23^ (Figure 1, Protocol 3) and those used for rescue of the *st63* hypomorphic allele (48–72 hpf, 10 μM)^22^ (Figure 1, Protocol 1; Figure S3).

**FIGURE 3:**
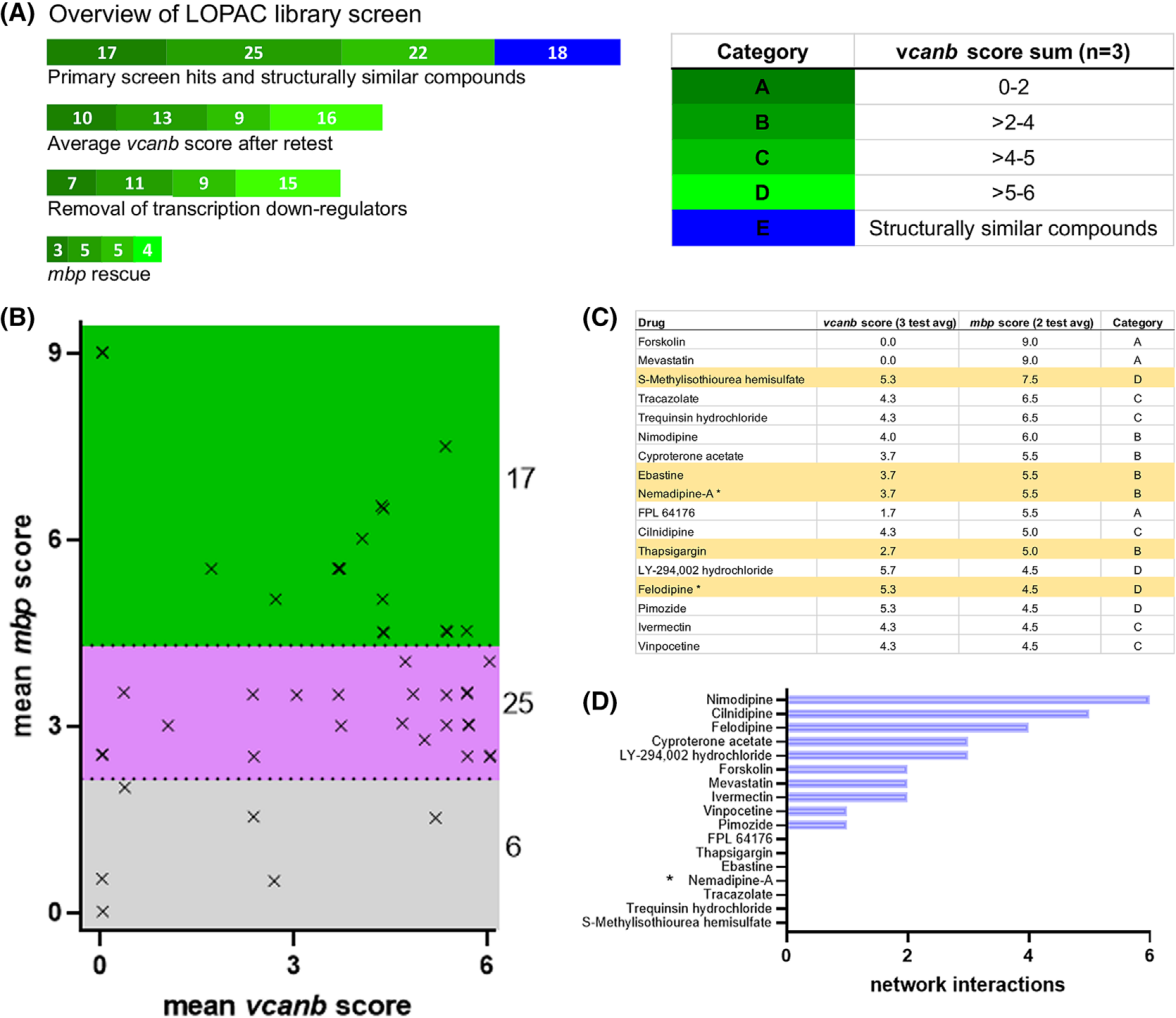
A counter screen reveals 17 hit compounds that can also rescue *mbp* expression in *adgrg6* ^*tb233c−/−*^ mutant embryos. (A) Overview of LOPAC library screen results. Hit compounds were grouped according to the strength of *vcanb* rescue according to the values in the table; dark green indicates the lowest *vcanb* score (category A; best rescue). The 18 compounds in blue correspond to the structurally similar compounds that were also retested. (B) Scatter plot of *vcanb* and *mbp* assay scores for the 48 *vcanb* assay hits. Jitter (noise) introduced to improve visualization. Green, final hits (17); magenta, *vcanb‐*only hits (25); grey, compounds that decreased *mbp* expression (false positives; 6). (C) Table of *vcanb* (3‐test mean score, nine embryos screened) and *mbp* (2‐test mean score, six embryos screened) assay scores for final 17 *mbp* hit compounds. Compounds only present in the LOPAC library are highlighted in yellow. Note that felodipine and nemadipine‐A were included in the E category (*) and were previously tested as part of the extended dihydropyridine cluster.^23^ (D) Number of structural similarity connections of individual hit compounds with other compounds in the network analysis.

Using this optimized assay, we tested the 48 hit compounds from the *vcanb* primary screen for their ability to increase *mbp* expression around the PLLg in *adgrg6* ^*tb233c−/−*^ hypomorphic mutant embryos, according to the scoring scheme shown in Figure 1. This identified 17 compounds (35% of *vcanb* assay hits; 1.3% of the LOPAC library) as *mbp* assay hits (total 2‐test average score ≥4.5) (Figure 3; Table S1). The *mbp* assay hits are a subgroup of compounds most likely to modulate Adgrg6 pathway activity, as they mediate a measurable rescuing effect on both transcriptional readouts (down‐regulating *vcanb* and up‐regulating *mbp*). There were 25 *vcanb‐*specific hits (52%) that elicited no clear effect on *mbp* expression at the concentration used. These compounds may target the receptor pathway in the ear specifically, or might exhibit reduced efficiency in reaching or penetrating Schwann cells in the PNS. This group includes some potent down‐regulators of *vcanb* (danazol, IC 261, oxaprozin, auranofin, dofetilide, and 7‐chloro‐4‐hydroxy‐2‐phenyl‐1,8‐naphthyridine, all scoring ≤1 in the *vcanb* assay). Such compounds should not be discounted as potential modulators of the Adgrg6 signalling pathway (Table S1). Critically, the *mbp* assay also allows identification of false‐positive compounds that caused the down‐regulation of both *vcanb* and *mbp*; 6 (13%) such compounds were found and eliminated from further analysis (Table S1).

### 2.4 Confirmation of hit compounds across multiple library screens and different assays

**FIGURE 4:**
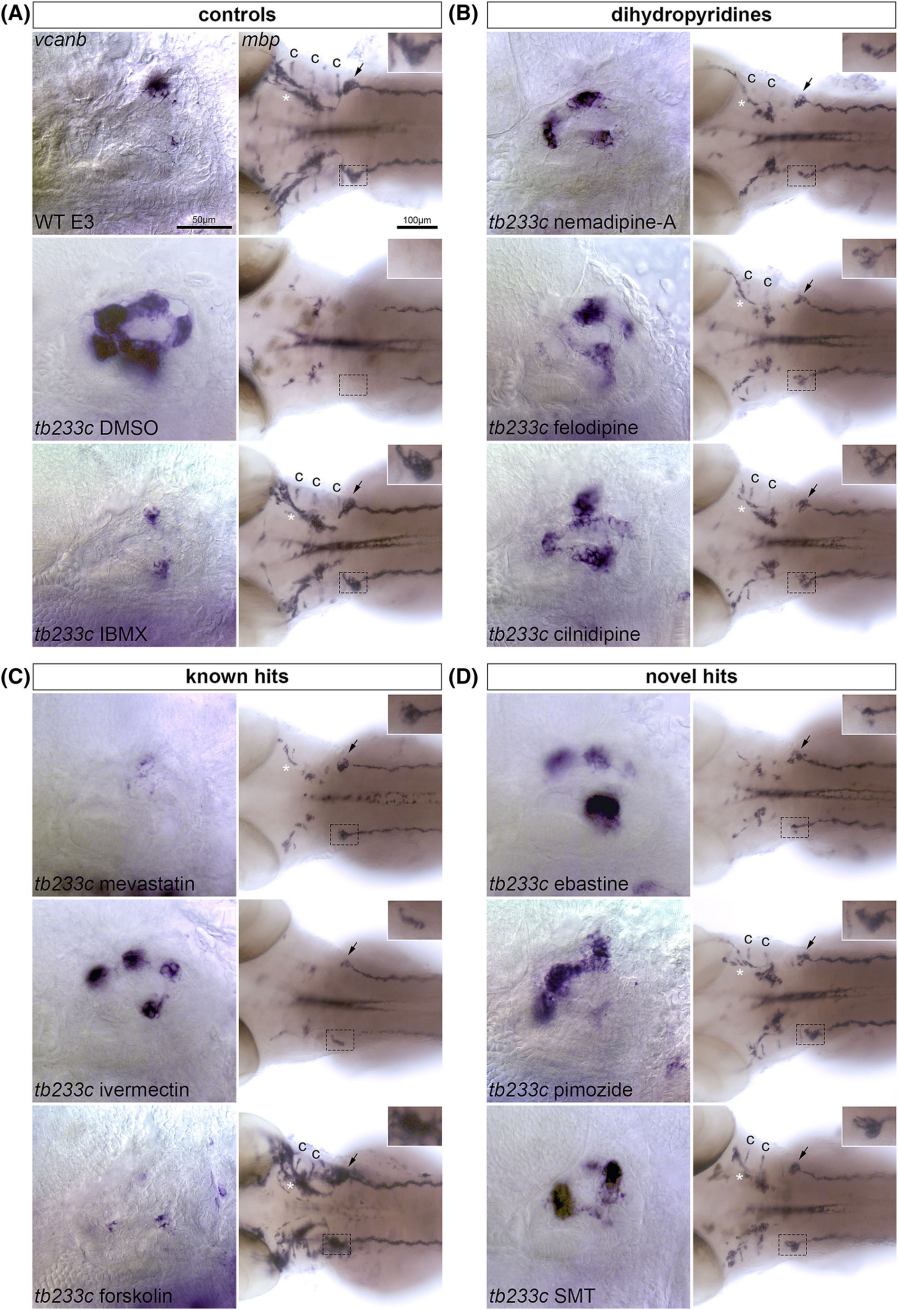
Hit compounds that mediate rescue of both *vcanb* and *mbp* expression in mutant embryos, and are possible modulators of the Adgrg6 pathway. Lateral images of the inner ear at 90 hpf stained for *vcanb* and dorsal images of embryos at 78 hpf stained for *mbp*. Anterior to the left in all images. (A) Images of control group embryos, including wild types incubated in E3 and *adgrg6* ^*tb233c−/−*^ mutants incubated in DMSO or IBMX (positive control). IBMX restores wild‐type levels of *vcanb* (inner ear) and *mbp* (PLLg) expression in *adgrg6* mutant embryos. The dotted rectangle marks the region of interest (ROI) enclosing the left PLLg, enlarged in the inserts in the top right of each *mbp* panel. (B–D) Images of *adgrg6* ^*tb233c−/−*^ mutants treated with dihydropyridine hit compounds from the LOPAC screen (B), previously known hit compounds from the Spectrum and Tocris screen also identified in the LOPAC screen (C) or novel hit compounds from the LOPAC screen (D). Hits mediate partial or full restoration of *vcanb* expression in the inner ear, and *mbp* expression around the PLLg. Note the over‐expression of *mbp* in the PNS of forskolin‐treated embryos (C, lower panels). Compounds were tested at 25 and 15 μM in the *vcanb* and *mbp* assays, respectively. The IBMX control was tested at 100 and 50 μM in the *vcanb* and *mbp* assays, respectively. Arrow, *mbp* expression restored around the PLLg; asterisk, expression restored along the supraorbital lateral line nerve; c, expression restored along nerves innervating the inner ear cristae.

Of the 17 *mbp* assay hits from the LOPAC screen, 12 compounds were previously known hits from the Tocris or Spectrum screens^23^ (Figure 3C). Forskolin and mevastatin were able to mediate restoration of both *vcanb* and *mbp* to wild‐type expression levels in *adgrg6* ^*tb233c−/−*^ hypomorphic mutant embryos (Figures 3 and S1); in the case of forskolin, *mbp* expression appeared up‐regulated in comparison with expression levels in wild‐type embryos (Figure 4). Mevastatin was recognized as a hit in the previous *vcanb* assay screen,^23^ but was toxic to embryos at concentrations above 25 μM. Four compounds are dihydropyridines (cilnidipine, nimodipine, nemadipine‐A, and felodipine). Two of these (cilnidipine, nimodipine) were also identified as hits in the Tocris or Spectrum libraries, whereas nemadipine‐A and felodipine were used successfully to test functional predictions from a network cluster analysis.^23^ The reidentification of all 12 hits from the LOPAC library demonstrates the reproducibility of the screening assay. Five compounds were LOPAC‐specific (Figure 3C; yellow shading; Table S1). Within this subgroup, three compounds (ebastine, S‐methylisothiourea hemisulfate [SMT], and thapsigargin) were structurally diverse from any molecules previously screened; these hits accounted for 1% (3/275) of the structurally novel compounds present in the LOPAC library.

Because initial hits were selected using the otic *vcanb* assay, the screening protocol does not identify compounds that can rescue only the myelination defect, but not the otic defect, in *adgrg6* mutant embryos. Apomorphine, a candidate modulator for Adgrg6 identified by Bradley et al. (2019), is present in the LOPAC, Spectrum and Tocris libraries but was not identified as a *vcanb* assay hit in any of our primary screens. Bradley et al. (2019) initially identified apomorphine as a weak rescuer of *mbp:gfp* transgene expression in *adgrg6* ^*st63−/−*^ hypomorphic missense mutant embryos at 10 μM, and demonstrated its dose‐dependent partial rescue of *mbp* mRNA expression; it was most effective, but also toxic, at 100 μM.^22^ We tested the ability of apomorphine to rescue the *adgrg6* ^*tb233c−/−*^ hypomorphic allele in both the *vcanb* and optimized *mbp* expression assays. Treatment at 50 μM, but not 25 μM, was able to reduce otic *vcanb* expression (Figure S4). A partial rescue was also observed in the *mbp* assay, although this was not significant at 10 and 15 μM (Figure S4). These data suggest that the *vcanb* in situ hybridization screen is more stringent than the *mbp:gfp* fluorescence screen in the initial identification of hit compounds. However, we can also confirm the rescuing effect of apomorphine at higher concentrations using the *vcanb* and *mbp* expression assays with the *tb233c* allele.

### 2.5 Testing selected hit compounds (ivermectin, ebastine) in dose–response assays

We selected two *mbp* assay hits, ivermectin and ebastine, as initial candidates for further analysis. Both compounds were tested in a concentration series ranging from 2.96 to 50.6 μM (dilution factor 1.5) to determine their therapeutic window, and general observations were made to assess their toxicity.

**FIGURE 5:**
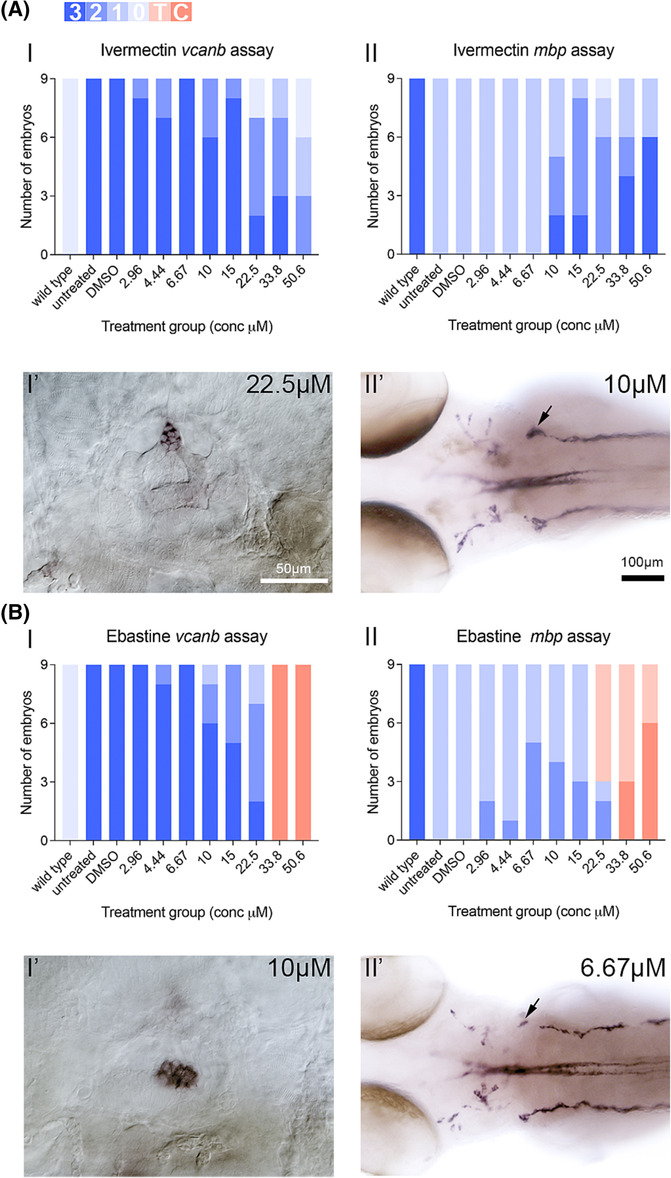
Performance of selected hits in dose–response assays on *adgrg6* ^*tb233c−/−*^ mutants. Ivermectin (A) and ebastine (B) were tested in a 1.5‐fold dilution series on groups of nine *adgrg6* ^*tb233c−/−*^ hypomorphic mutant embryos. Charts show the number of mutant embryos (columns 2–11) that scored 0, 1, 2, or 3 in the *vcanb* and *mbp* assays, in comparison with untreated wild‐type embryos (column 1). The blue shades represent the in situ hybridization staining score for each gene. For *vcanb*, a pale shade indicates full rescue (reduction) of otic expression to wild‐type levels (*vcanb* score 0; see scale), whereas the darkest shade indicates no rescue (*vcanb* score 3). For *mbp*, the darkest shade indicates full rescue (restoration) of expression around the PLLg (*mbp* score 3), and pale shades no rescue (*mbp* scores 1 or 0). Toxic effects are highlighted in red (pale, toxic (T); dark, corrosive (C; no embryos in well)). (A) Example images show lateral view of an ear from a mutant embryo treated with 22.5‐μM ivermectin in the *vcanb* assay (A I′, score 0), and dorsal view of a mutant embryo treated with 10‐μM ivermectin in the *mbp* assay (A II′, score 2). (B) Example images show lateral view of an ear from a mutant embryo treated with 10‐μM ebastine in the *vcanb* assay (B I′, score 1) and dorsal image of an embryo treated with 6.67‐μM ebastine in the *mbp* assay (B II′, score 2). Arrows mark restoration of *mbp* expression around the PLLg.

Ivermectin, a macrocylic lactone used as an antiparasitic in both veterinary and human medicine, was independently and reproducibly identified as a hit in all three libraries (a strong *vcanb* hit in the LOPAC and Spectrum collections, and a weaker hit in the Tocris library^23^; Figure S1). Ivermectin acts as an allosteric agonist of glutamate‐gated chloride channels in the nanomolar range, and can bind to, activate or modulate a variety of transmembrane receptors at higher concentrations.^29^, ^30^, ^31^, ^32^ It was ineffective at rescuing the protein‐truncating *adgrg6* allele *fr24*,^23^ making it a candidate for binding directly to the Adgrg6 receptor. Ivermectin mediated a dose‐dependent decrease in *vcanb* mRNA expression in *adgrg6* ^*tb233c−/−*^ hypomorphic embryos; it was most effective at 50.6 μM, with all embryos displaying some reduction in otic *vcanb* expression (Figure 5A). Ivermectin was also effective at rescuing *mbp* expression around the PLLg at concentrations ≥10 μM (Figure 5B).

Ivermectin has previously been reported to be both cardiotoxic and ototoxic in the zebrafish.^33^, ^34^ In our assays, ivermectin had some toxicity above 1 μM, reducing embryo movement (not shown). At 50 μM, ivermectin treatment resulted in pericardial oedema and a failure to inflate the swimbladder (Figure S5). However, we found no evidence of severe ototoxicity, even at higher concentrations: lateral line neuromasts appeared relatively normal in *Tg (pou4f3:GFP)* embryos after treatment with 100 μM ivermectin (Figure S5).

Ebastine, a piperidine derivative, and a widely marketed antihistaminic drug, is an inverse agonist for the histamine H1 receptor, a rhodopsin‐class GPCR.^35^, ^36^ Ebastine was one of three LOPAC‐specific hits, and is not closely structurally related to any other compound in the three libraries (Figures 3C and S1). Although ebastine was toxic above 25 μM, it rescued otic *vcanb* expression over a restricted concentration range of 15–22.5 μM (Figure 5A), and gave a partial rescue of *mbp* expression at concentrations between 2.96 and 15 μM (Figure 5B). Interestingly, a number of other compounds that are structurally unrelated to ebastine, but which also function to block H1 receptor activity, were also isolated as hit compounds in the primary screen. These include cyproheptadine hydrochloride (a strong *vcanb* hit from all three libraries) and flunarizine dihydrochloride (a strong *vcanb* hit from the LOPAC and Tocris libraries) (Figure S1). This demonstrates the value of grouping hit compounds by biological function as well as chemical structure.

## 3 DISCUSSION

Adhesion GPCRs are key components of signalling pathways involved in development and disease and have huge potential for drug discovery.^37^ In this study, we set out to identify potential modulators of the Adgrg6 signalling pathway using a previously developed in vivo assay. Utilizing transcriptional readouts of *vcanb* and *mbp* as measures of Adgrg6 signalling activity in an *adgrg6* hypomorphic zebrafish mutant, we have identified 48 compounds that consistently downregulated otic *vcanb* expression in *adgrg6* ^*tb233c−/−*^ embryos, 17 of which also restored *mbp* expression in Schwann cells around the posterior lateral line ganglion (PLLg) in an optimized *mbp* assay.

The *adgrg6* screening regime displayed a high degree of reproducibility. Screening of the LOPAC library enabled blind retesting of previously identified hits that were also represented in the Spectrum and Tocris libraries. Compounds such as ivermectin and mevastatin, along with the dihydropyridine nimodipine, were identified as hits in all three libraries and confirmed with different *mbp* assay conditions. Within the LOPAC screen, consistent scoring was also evident between multiple retests for both *vcanb* and *mbp* scores. The scoring criteria for the initial primary screen hits were less stringent than for the retests to increase the likelihood of identifying all potential hit compounds; however, this did result in a higher number of false positives after retesting.

At the screening concentration tested (25 μM), 25 compounds were only able to rescue otic *vcanb* expression, but not *mbp* (Table S1). Although some of these compounds might mediate rescue of the *mbp* phenotype at different concentrations, their differing activities in the two tissues of interest also illustrate potential differences in Adgrg6 signalling pathways within the ear and those in Schwann cells. In the inner ear, Adgrg6 is required for the epithelial contact and fusion events that lead to formation of the semicircular canal pillars, and is expressed on both sides of fusing epithelial projections.^10^ In Schwann cells, Adgrg6 is thought to be activated by contact with an axon, triggering a cascade of gene expression that drives Schwann cell differentiation beyond the promyelinating stage.^3^ It is also important to consider that *vcanb*‐specific compounds may act directly to modulate *vcanb* expression. Such compounds could hold therapeutic potential as overexpression of *versican* is associated with inflammation and cancer progression^38^, ^39^ (and references within).

### 3.1 Candidate Adgrg6 pathway modulators

Adgrg6 is thought to signal through a canonical Gα_s_/adenylyl cyclase/cAMP/PKA pathway to regulate myelination of the PNS by Schwann cells.^1^, ^3^ A selection of compounds that are known to mediate elevation of cytoplasmic cAMP levels were identified as hits, including the adenylyl cyclase agonists colforsin and forskolin, and the phosphodiesterase inhibitors IBMX, vinpocetine and trequinsin hydrochloride (Diamantopoulou et al.^23^ and this work). These findings provide further support for the interpretation that Adgrg6 signals through Gα_s_, but potentially also through Gα_q/11_ and Gα_12/13_, as these pathways involve cGMP signalling, which is also elevated by inhibition of phosphodiesterases.^40^, ^41^ Furthermore, identification of such compounds provides proof of concept for the screening assays, as the phenotypic rescuing effects of forskolin and IBMX—on various *adgrg6* zebrafish mutant alleles—have been well documented^3^, ^10^, ^23^.

The LOPAC hits confirm and extend the existing collection of putative Adgrg6 modulators identified in previous screens. The follow‐up work on ivermectin and ebastine described here illustrates some of the considerations involved in the further testing of primary screen hits. There are many additional compounds of interest that could form the basis of future studies. Functional links between hit compounds are highlighted by the repeated identification of histamine H1 receptor antagonists (this work) and L‐type calcium channel interactors (Bradley et al.,^22^ Diamantopoulou et al.,^23^ and this work). The synthetic steroid danazol was also reproducibly identified as a hit (Diamantopoulou et al.^23^ and this work). Steroids are good candidates for molecules that interact directly with the receptor; the steroidal core of glucocorticoids, for example, has been shown to bind to and activate the human aGPCR ADGRG3/GPR97.^42^ Molecules of the steroid‐like gedunin family, including 3‐α‐deoxygedunin (3‐α‐DOG), are not represented in the LOPAC library, but remain very promising candidates as Adgrg6 interactors^23^ (Figure S1).

Zebrafish screens have proved increasingly successful at identifying drugs that can then translate to use in other species.^43^, ^44^, ^45^ The in vivo screening strategy described here is equally suitable for libraries covering a wide range of chemical space, or for those tailored to specific families of compounds. It has the added advantage of screening for rescue of the *adgrg6* mutant phenotype in two very different tissue contexts, strengthening support for candidate compounds with specific agonistic activity for the Adgrg6 pathway. Further testing on protein‐truncating alleles provides an effective way to differentiate hit compounds and prioritize those likely to bind directly to the receptor.^22^, ^23^

In summary, we have identified a number of promising hits from the combined screens of the LOPAC, Spectrum and Tocris libraries, several of which fall into structurally related groups represented by two or more hit compounds (Diamantopoulou et al.^23^ and this work). Future structure–activity relationship (SAR) analysis could use information from hit compounds to select or design molecules with improved characteristics, including increased specificity and reduced toxicity. Precedent for this approach is illustrated through the testing of families of structurally related compounds to those identified as screening hits, which resulted in the identification of additional dihydropyridines^23^ or aporphine alkaloids^22^ with rescuing activity in *adgrg6* hypomorphic mutants. Once a suitable molecule is found, further work could include identification of the binding pocket in the Adgrg6 receptor for the compound of interest, and a determination of its mechanism of action.

## 4 MATERIALS AND METHODS

### 4.1 Animals

All zebrafish strains were raised and maintained in the aquarium facility at the University of Sheffield, in accordance with UK Home Office licence regulations. Adult zebrafish were housed in mixed‐sex groups in plastic tanks (Tecniplast) with circulating water maintained at 28.5°C on a 14‐h light/10‐h dark cycle. Embryos were harvested into E3 medium (5‐mM NaCl, 0.17‐mM CaCl_2_, 0.33‐mM MgSO_4_) at 28.5°C, sorted into groups of approximately 50 in plastic 10 mm Petri dishes, and staged according to Kimmel et al.^46^ Homozygous *nacre* (*mitfa* ^*w2−/−*^; ZDB‐GENO‐990423‐18) embryos lacking melanophores^47^ were employed as wild‐type controls in drug screening experiments, for the ease of visualizing in situ hybridization patterns. The zebrafish *adgrg6* mutant line used was the hypomorphic missense allele *tb233c* (I963N; ZDB‐ALT‐980203‐351).^9^, ^10^ Figure 1A shows how this allele compares with alleles used in previous studies^22^, ^23^ and mentioned in the text. The transgenic line used to assess hair cell viability (Figure S5) was *Tg (pou4f3:GAP‐GFP)* (ZDB‐TGCONSTRCT‐070117‐142).^48^

### 4.2 Compound storage and preparation

The drug screening assay was performed using the Sigma Library of Pharmacologically Active Compounds (LO4200, LOPAC^1280^, Sigma‐Aldrich), comprising 1280 compounds dissolved in DMSO arrayed across 16 plates. V‐bottom 96‐well microtitre plates (Matrix) containing 2.5 μl of compound in each well at 2.5 mM (or 1.5 mM) were prepared for screening. Initial hit compounds and additional compounds of interest were cherry‐picked from the Sigma LOPAC master plates (5‐mM concentration) and used for retests and counter screening assays. Compounds selected for follow‐up experiments were purchased separately: ivermectin (Sigma I8898) and ebastine (Sigma E9531).

### 4.3 Assay development

Wild‐type (*mitfa* ^*w2−/−*^) and *adgrg6* ^*tb233c−/−*^ homozygous mutant embryos were either dechorionated by hand with forceps or treated with pronase and washed extensively before drug incubation. For the *vcanb* assay, embryos had a final overnight incubation at 20°C to slow down their development to reach the equivalent of 60 hpf in the morning (estimated in accordance with Kimmel et al.^46^). For *mbp* screening experiments, the embryos were kept at 28.5°C after dechorionation to develop to 54 hpf prior to drug administration. For the drug screening assay plate, 2.5 μl of control compounds (2.5 mM) were administered in columns 1 and 12, including 1% DMSO as a negative control, and 5‐ to 10‐mM 3‐isobutyl‐1‐methylxanthine (IBMX) as a positive control; 80 library compounds were arrayed in columns 2–11; 247.5 μl of fresh pre‐warmed (28.5°C) E3 was added to each well and transferred to a receiver plate to give a final compound concentration of 15–25 μM for screening. At the appropriate stage of development, three embryos were aliquoted into each well of a MultiScreen 96‐well mesh‐bottomed plate (100 µm; Millipore) in E3 medium. The mesh plates were then transferred to the receiver (assay) plate of compounds and incubated at 28.5°C for 24–30 h, depending on the experiment. Following this incubation, mesh‐bottomed plates containing embryos were transferred into 4% (v/v) paraformaldehyde (PFA) and stored at 4°C overnight before bleaching with 3% H_2_O_2_/0.5% KOH in E3 medium for approximately 20 min to remove pigment. Embryos were stored in 100% methanol at −20°C prior to in situ hybridization. For toxicity assays, larvae were treated as above in 96‐well plates (three embryos per well).

### 4.4 In situ hybridization

In situ hybridization was performed in the MultiScreen mesh‐bottomed multiwell plates using the Biolane HTI 16 V in situ robot (Intavis). Digoxigenin‐labelled RNA probes were prepared for *vcanb* ^49^ and *mbp* (*mbpa*).^50^ The whole‐mount in situ hybridization protocol followed standard procedures^51^ with modifications for higher throughput.^52^

### 4.5 Hit selection

Stained embryos were scored manually using the scoring system shown in Figure 1. Briefly, for *vcanb* expression, scores were 0 for complete rescue of expression to low (wild‐type) levels, 1 or 2 for intermediate levels of expression, and 3 for no reduction of expression (no rescue). The individual scores for three embryos in a microplate well were summed to give a value between 0 and 9. Compounds with a summed score of <6 were taken forward for retests. The average score for each compound across the three retests was taken as the final score.

Expression of *mbp* around the posterior lateral line ganglion (PLLg) was scored for individual embryos as follows: 3 for complete restoration of expression to wild‐type levels (full rescue), 2 for intermediate rescue, 1 for no change in expression, and 0 for down‐regulated expression of *mbp* around the posterior lateral line ganglion (PLLg) and in the PNS overall (Figure 1). The individual scores for three embryos in a microplate well were summed to give value between 0 and 9. The threshold for ability to rescue *mbp* expression was set as a total average score of ≥4.5.

### 4.6 Dose response assay

Wild‐type and *adgrg6* ^*tb233c−/−*^ mutant zebrafish embryos were raised to the equivalent stage of 54–60 hpf (see above) prior to drug administration in E3. At the appropriate developmental stage, embryos were dechorionated before being placed in a 24‐well plate (10 embryos/well in 400 μl of E3). A 1.5‐fold dilution series of each compound was prepared in DMSO before diluting in E3 and adding 100 μl directly to the plate wells for testing to give a final volume of 500 μl; DMSO was kept at 1% (v/v) per well.

### 4.7 Imaging

Whole‐well images of the screening assay plates were taken with a Nikon AZ100 microscope with an automated stage (Prior Scientific), using NIS‐Elements Extended Depth of Focus software (Nikon) to create a compressed in‐focus image. Individual stained embryos were mounted in 70% glycerol and imaged using an Olympus BX51 microscope with a C3030ZOOM camera and CellB software, or a Micropublisher 6 camera and Ocular software. All lower magnification micrographs are dorsal views with anterior to the left; all higher magnification images of the ear are lateral views with anterior towards the left and dorsal towards the top. Images of ivermectin‐treated embryos in Figure S5 were taken with a Zeiss AX10 Zoom V16 microscope and Zen software. Embryos were mounted laterally and dorsally using a Stampwell agarose mould (Idylle).

### 4.8 Chemoinformatics and data visualization

Library compound structures were represented as SMILES and processed as previously described.^23^ A dendrogram of the LOPAC library compounds, based on the similarity matrix between all compound pairs, was generated using the SciPy library (http://www.scipy.org/, accessed 06 November 2018). The Tanimoto coefficient was used to calculate the compound similarity^53^ using the scikit‐learn library.^54^ To create the compound cluster network, a dendrogram of the LOPAC, Spectrum and Tocris Total libraries was transformed into an adjacency matrix using a threshold value of 0.5; that is, compounds with a similarity value >0.5 are connected with an edge. The network visualization was created using Cytoscape^55^ and can be accessed using the link (https://adlvdl.github.io/visualizations/network_whitfield_lopac_tocris/index.html).

### 4.9 Image and statistical analysis

Staining from in situ hybridization images was measured in Fiji,^56^ as previously described.^23^ Statistical analyses were performed using GraphPad Prism version 8 for Mac (GraphPad Software, La Jolla California USA, www.graphpad.com).

### 4.10 Ethics

The study was conducted in accordance with the Basic & Clinical Pharmacology & Toxicology policy for experimental and clinical studies.^57^ All animal work was performed under licence from the UK Home Office.

## CONFLICT OF INTEREST STATEMENT

AA and GRW are employees and shareholders of Sosei Heptares.

## LICENCING

For the purpose of open access, the author has applied a Creative Commons Attribution (CC BY) licence to any Author Accepted Manuscript version arising.

## Supporting information


**Table S1.** List of the 48 hit compounds from the LOPAC primary screen.
**Figure S1.** Cluster analysis of LOPAC, Spectrum and Tocris Total compound library *vcanb* primary screening results.
**Figure S2.** Comparison of scores between different libraries and between retests within the LOPAC library.
**Figure S3.** Testing alternative *mbp* assay protocols in *adgrg6tb233c−/−* mutant embryos.
(A, B) Three different protocols were assessed to identify the optimum *mbp* screening conditions. (A) Bright‐field images of *mbp* expression in embryos following compound incubation under assay conditions that displayed some or complete rescue of the *adgrg6tb233c−/−* mutant phenotype; dorsal views with anterior to the left. The dotted rectangle (150 ´ 100 pixels) marks the region of interest (ROI) enclosing the left PLLg, enlarged in the inserts in the top right of each panel, and quantified in (B). Arrows indicate *mbp* expression in Schwann cells around the right PLLg; asterisk in the top row (48–72 hpf) indicates fixation of embryos at 96 hpf following 24‐hour incubation in E3. (B) Area of *mbp* expression as a percentage of the total ROI illustrated in (A). Each data point represents staining around a single PLLg (*n* = 12 ganglia, *N* = 6 embryos per treatment). Error bars, 95% confidence interval; ns, *p* ≥ 0.05; *p = 0.01–0.05; **p = 0.001–0.01; ****p < 0.0001. One‐way ANOVA with Tukey's post‐test correction for multiple comparisons.
**Figure S4.** Apomorphine mediates partial rescue of the *mbp* and *vcanb* phenotype in *adgrg6tb233c−/−* mutant embryos.
**Figure S5.** Performance of ivermectin in zebrafish toxicity assays. (A) Treatment with 50 μM ivermectin for 44 h gives a partial rescue of ear swelling and fusion of semicircular canal projections in homozygous hypomorphic *adgrg6tb233c−/−* mutants. Black arrowheads mark the swollen ear in control (untreated) mutants; white arrowheads mark partial rescue of the ear phenotype in two individual mutant embryos. Treatment at this dose is cardiotoxic; the asterisk marks pericardial oedema, not present in controls. (B) No gross changes in GFP fluorescence (inverted GFP channel) in sensory hair cells of the maculae in the ear (yellow arrowhead) and lateral line neuromasts (blue arrowhead) were observed after treatment with 100 μM ivermectin for 44 h. Insets show enlargements of representative individual posterior lateral line neuromasts, with healthy hair cells visible.

## Data Availability

The data that support the findings of this study are available from the corresponding authors upon reasonable request.
