# Supplementary material for: A screen of pharmacologically active compounds to identify modulators of the Adgrg6/Gpr126 signalling pathway in zebrafish embryos
Source: Basic Clin Pharmacol Toxicol. 2023 Jul 16;133(4):364–77. doi: 10.1111/bcpt.13923 (PMC10952222; doi:10.1111/bcpt.13923)
Supplement: Supplementary file 1 — Table S1. List of the 48 hit compounds from the LOPAC primary screen. Figure S1. Cluster analysis of LOPAC, Spectrum and Tocris Total compound library vcanb primary screening results. Figure S2. Comparison of scores between different libraries and between retests within the LOPAC library. Figure S3. Testing alternative mbp assay protocols in adgrg6tb233c−/− mutant embryos. (A, B) Three different protocols were assessed to identify the optimum mbp screening conditions. (A) Bright‐field images of mbp expression in embryos following compound incubation under assay conditions that displayed some or complete rescue of the adgrg6tb233c−/− mutant phenotype; dorsal views with anterior to the left. The dotted rectangle (150 ´ 100 pixels) marks the region of interest (ROI) enclosing the left PLLg, enlarged in the inserts in the top right of each panel, and quantified in (B). Arrows indicate mbp expression in Schwann cells around the right PLLg; asterisk in the top row (48–72 hpf) indicates fixation of embryos at 96 hpf following 24‐hour incubation in E3. (B) Area of mbp expression as a percentage of the total ROI illustrated in (A). Each data point represents staining around a single PLLg (n = 12 ganglia, N = 6 embryos per treatment). Error bars, 95% confidence interval; ns, p ≥ 0.05; *p = 0.01–0.05; **p = 0.001–0.01; ****p < 0.0001. One‐way ANOVA with Tukey's post‐test correction for multiple comparisons. Figure S4. Apomorphine mediates partial rescue of the mbp and vcanb phenotype in adgrg6tb233c−/− mutant embryos. Figure S5. Performance of ivermectin in zebrafish toxicity assays. (A) Treatment with 50 μM ivermectin for 44 h gives a partial rescue of ear swelling and fusion of semicircular canal projections in homozygous hypomorphic adgrg6tb233c−/− mutants. Black arrowheads mark the swollen ear in control (untreated) mutants; white arrowheads mark partial rescue of the ear phenotype in two individual mutant embryos. Treatment at this dose is cardiotoxic; the asterisk marks [file BCPT-133-364-s001.docx]

# A screen of pharmacologically active compounds to identify modulators of the Adgrg6/Gpr126 signalling pathway in zebrafish embryos

**Supporting Information**

Anzar Asad^1^, Nahal O. Shahidan^1^, Antonio de la Vega de León^2^, Giselle R. Wiggin^3^, Tanya T. Whitfield^1^*^#^, Sarah Baxendale^1,4^*^#^

^1^ School of Biosciences, University of Sheffield, Sheffield, S10 2TN, UK

^2^ Information School, University of Sheffield, Sheffield, S1 4DP, UK

^3^ Sosei Heptares, Steinmetz Building, Granta Park, Cambridge, CB21 6DG, UK

^4^ Sheffield Zebrafish Screening Facility, School of Biosciences, University of Sheffield, Sheffield, S10 2TN, UK

*These authors contributed equally

^#^Corresponding authors:

Tanya T. Whitfield: [t.whitfield@sheffield.ac.uk](mailto:t.whitfield@sheffield.ac.uk)

Sarah Baxendale: s.baxendale@sheffield.ac.uk

**Table S1. List of the 48 hit compounds from the LOPAC primary screen.**

The 17 compounds that can rescue both *vcanb* and *mbp* expression in *adgrg6^tb233c-/-^* homozygous mutants are shown in green. The 25 compounds that rescued *vcanb* expression only are shown in blue. The 6 down-regulators of gene expression in both assays, counted as false-positives, are shown in orange. Dark shading indicates the 25 hits that were reproducibly identified in both the LOPAC and one or both of the Spectrum and Tocris libraries; pale shading indicates the 23 LOPAC-specific hits. *LOPAC-specific compounds previously shown to have rescuing activity ^1^. **Compounds represented in two or more libraries, but only identified as hits from the LOPAC library.

**
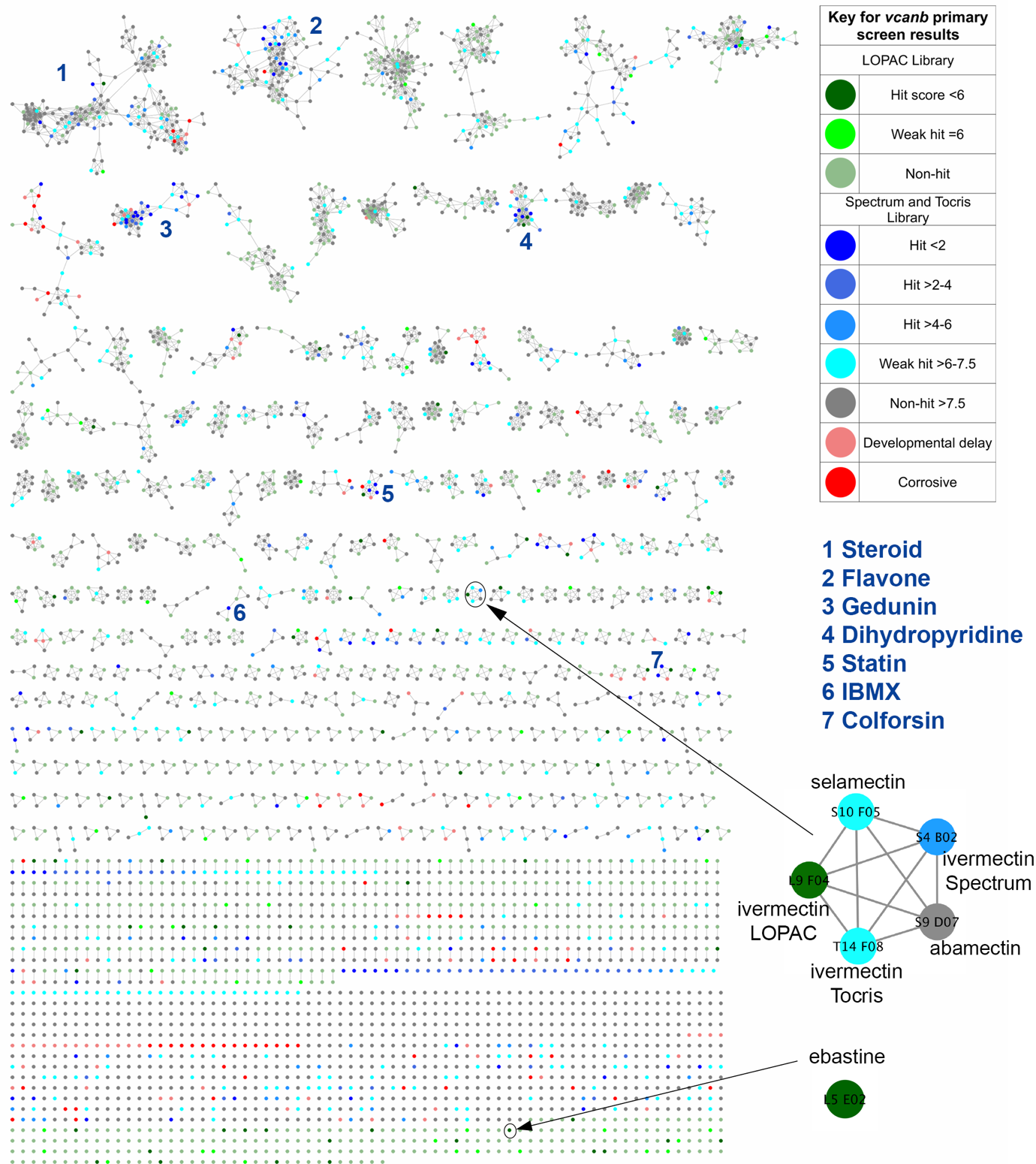
**

**Figure S1. Cluster analysis of LOPAC, Spectrum and Tocris Total compound library *vcanb* primary screening results.**

Image of the combined compounds of the LOPAC, Spectrum and Tocris Total libraries, clustered by structural similarity (Tanimoto coefficient; threshold$\geq$ 0.5). Hit scores are from the *vcanb* primary screen only. Compounds are shaded according to the key at the top right, and are labelled with a unique code starting with L (LOPAC), S (Spectrum) or T (Tocris), visible in the interactive version. Selected clusters containing multiple structurally-related hit compounds are numbered and listed on the right. Structurally unique compounds appear as individual dots at the bottom of the diagram. The two example hit compounds, ivermectin and ebastine, are highlighted. The ivermectin cluster also has a weak hit in the Tocris library, selamectin. Link to the interactive version: <https://adlvdl.github.io/visualizations/network_whitfield_lopac_tocris/index.html>.


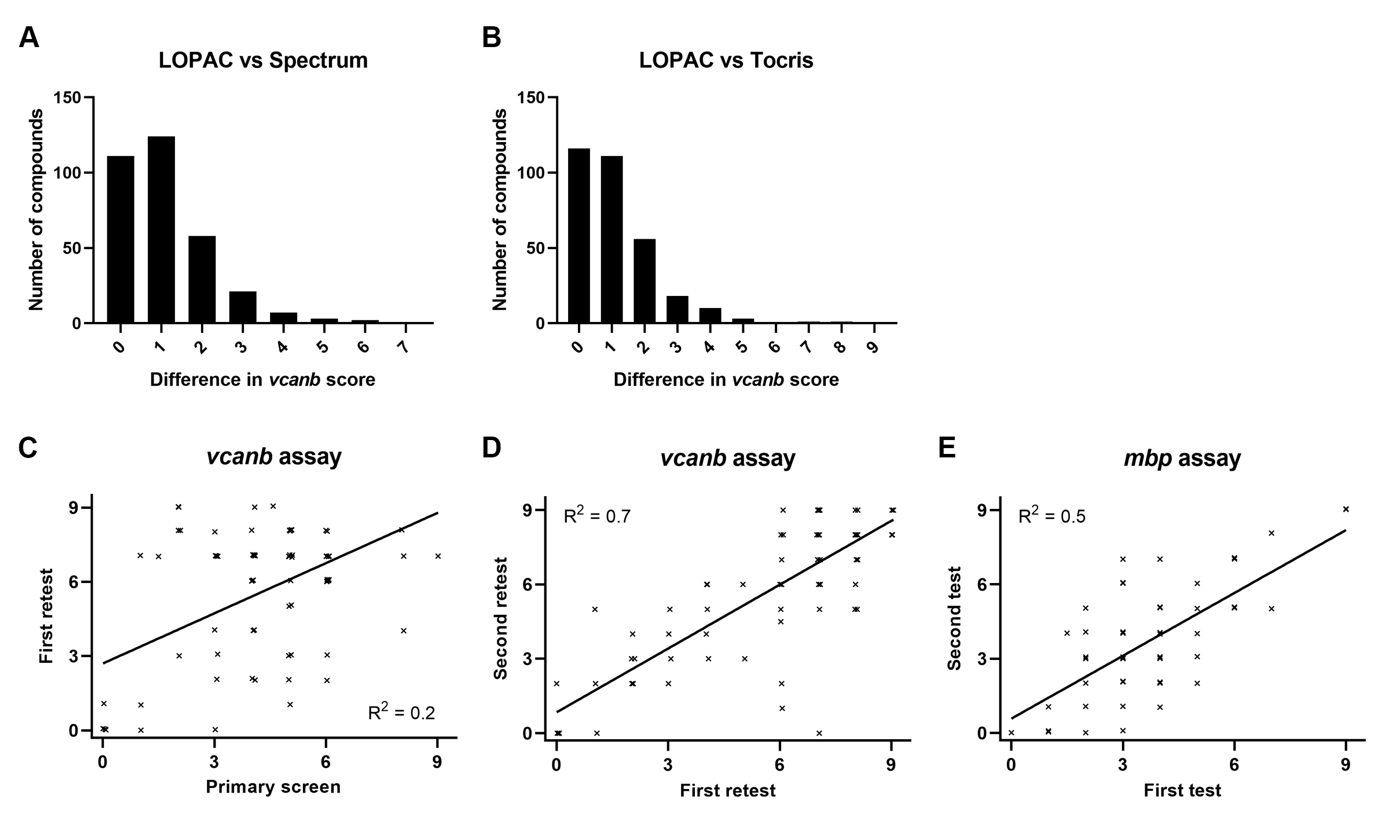


**Figure S2. Comparison of scores between different libraries and between retests within the LOPAC library.**

**(A, B)** Comparison of scores for the overlapping compounds between the LOPAC and Spectrum libraries (**A**; 326 compounds) and LOPAC and Tocris libraries (**B**; 316 compounds). The majority of compounds have the same score or differ by $\leq$2. (**C–E)** Comparison of scores from the LOPAC primary screen and retests. Correlation of scores from the primary screen and first retest (**C**) is low (*R*^2^ = 0.2; linear regression), as false-positive hits are excluded in the rescreen. By contrast, the first and second retest data for both the *vcanb* (*R*^2^ = 0.7) and *mbp* (*R*^2^ = 0.5) assays show a clear positive correlation (**D, E**).


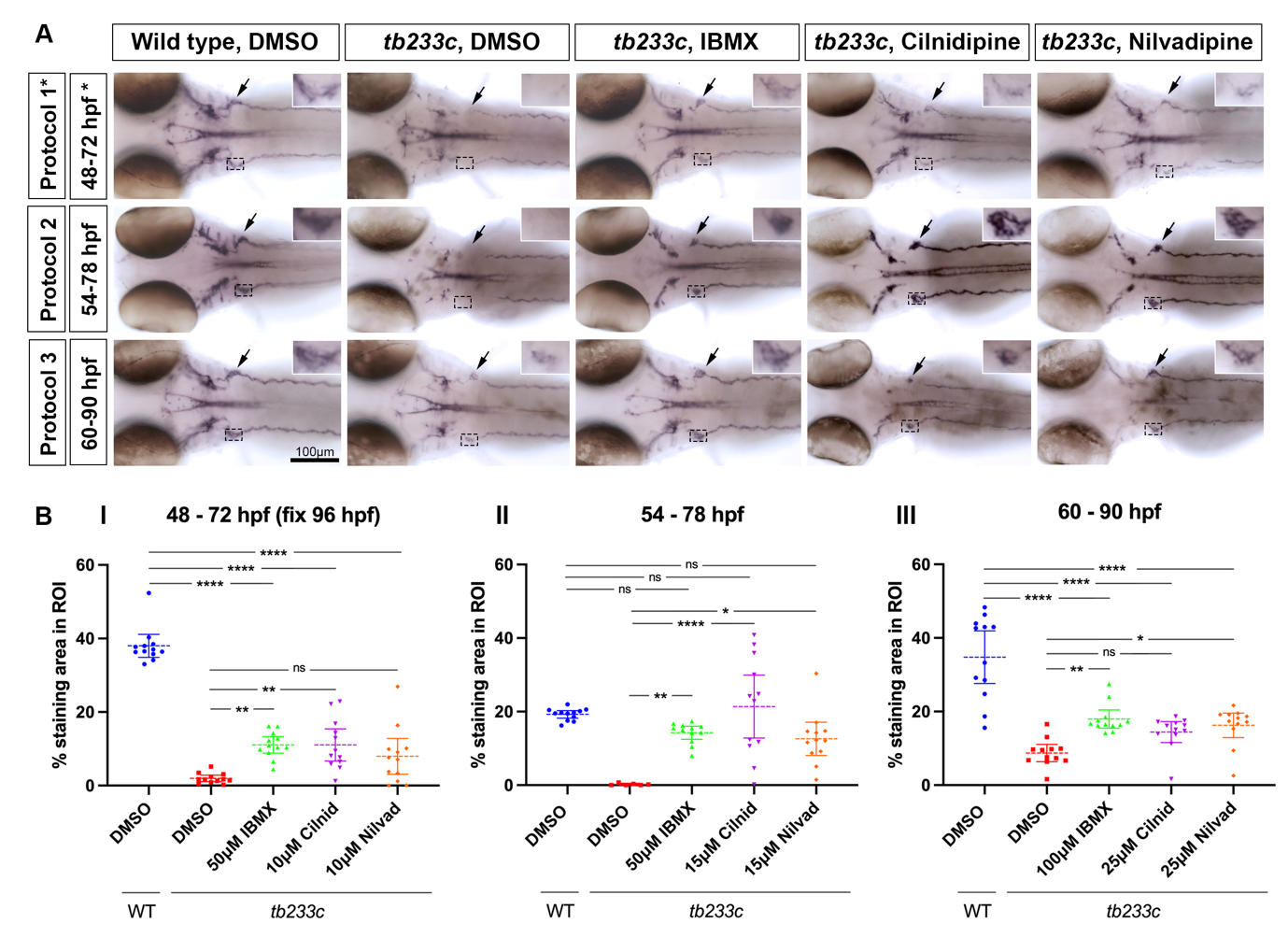


**Figure S3. Testing alternative *mbp* assay protocols in *adgrg6^tb233c-/-^* mutant embryos.**

**(A, B)** Three different protocols were assessed to identify the optimum *mbp* screening conditions. **(A)** Bright-field images of *mbp* expression in embryos following compound incubation under assay conditions that displayed some or complete rescue of the *adgrg6^tb233c-/-^* mutant phenotype; dorsal views with anterior to the left. The dotted rectangle (150 × 100 pixels) marks the region of interest (ROI) enclosing the left PLLg, enlarged in the inserts in the top right of each panel, and quantified in (**B**). Arrows indicate *mbp* expression in Schwann cells around the right PLLg; asterisk in the top row (48–72 hpf) indicates fixation of embryos at 96 hpf following 24-hour incubation in E3. **(B)** Area of *mbp* expression as a percentage of the total ROI illustrated in (**A**). Each data point represents staining around a single PLLg (*n*=12 ganglia, *N*=6 embryos per treatment). Error bars, 95% confidence interval; ns, *p* ≥0.05; **p* =0.01–0.05; ***p* =0.001–0.01; *****p* <0.0001. One-way ANOVA with Tukey’s post-test correction for multiple comparisons.


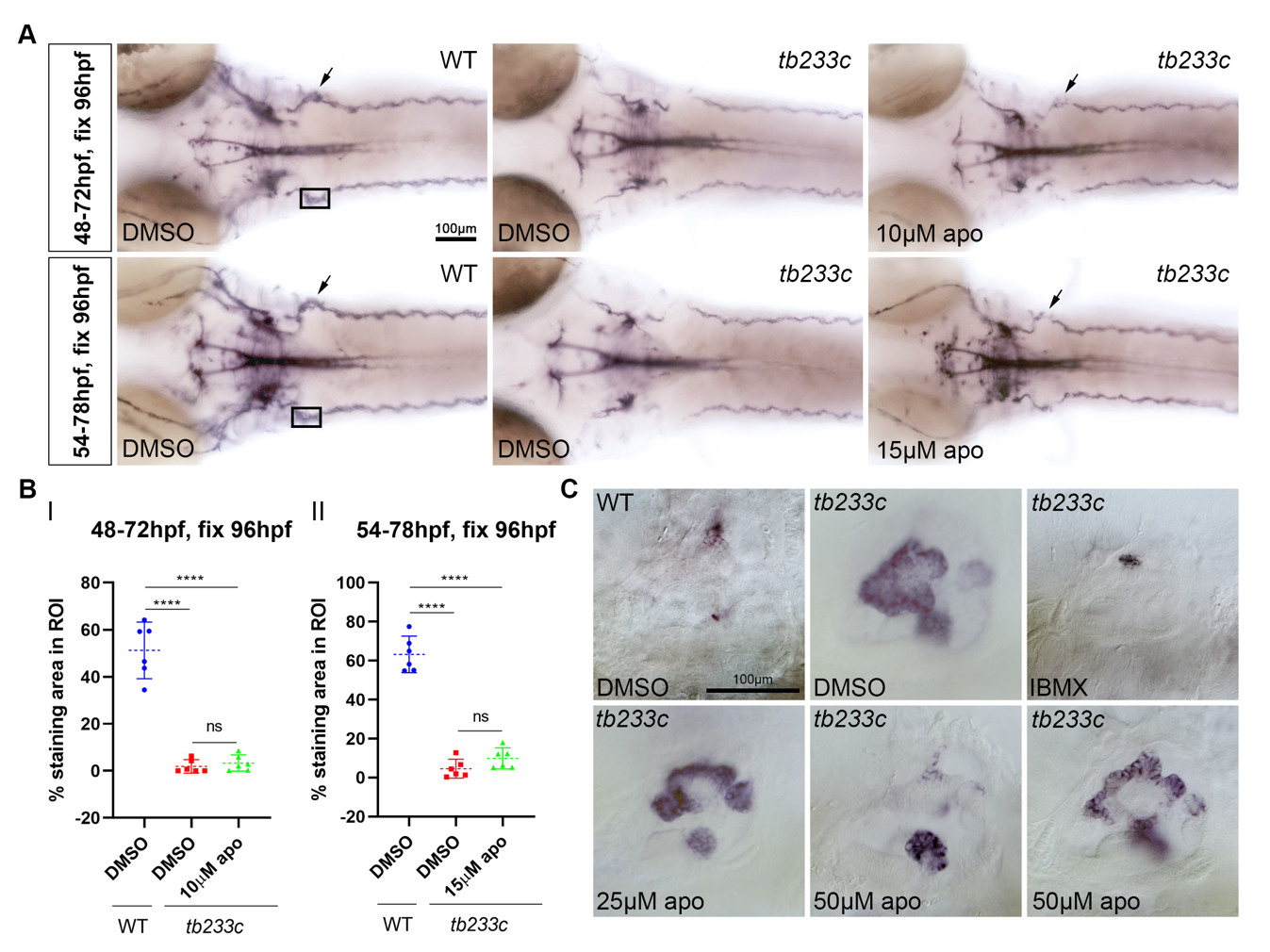


**Figure S4. Apomorphine mediates partial rescue of the *mbp* and *vcanb* phenotype in *adgrg6^tb233c-/-^* mutant embryos.**

**(A)** Bright-field images of *mbp* transcript expression in embryos following apomorphine (apo) incubation under assay conditions similar to those utilised by Bradley et al. (2019), top row, and those utilised in the current project, bottom row. Dorsal views; anterior to the left. Dotted rectangle (150 × 100 pixels) enclosing left PLLg of wild-type embryos illustrates region of interest (ROI) quantified in (B). Arrows indicate *mbp* expression in Schwann cells around the PLLg; all embryos were fixed at 96 hpf following a 24-hour incubation in E3. **(B)** Quantified area of *mbp* expression as a percentage of total ROI illustrated in (**A**). Each data point represents staining around a single PLLg (*n*=6 ganglia, *N=*3 embryos for each treatment). Although partial rescue was visible in the images in some embryos under both assay conditions (**A**, right hand panels), the thresholded and quantified staining area in apomorphine-treated embryos was not significantly different to DMSO controls (**B**). Error bars, 95% confidence interval; ns, *p* ≥0.05; *****p* <0.0001. One-way ANOVA with Tukey’s post-test correction for multiple comparisons. **(C)** Apomorphine failed to down-regulate *vcanb* expression in *adgrg6^tb233c-/-^* mutants at 25 μM, but showed partial rescue in some embryos at 50 μM (two examples illustrated, panels in lower right).


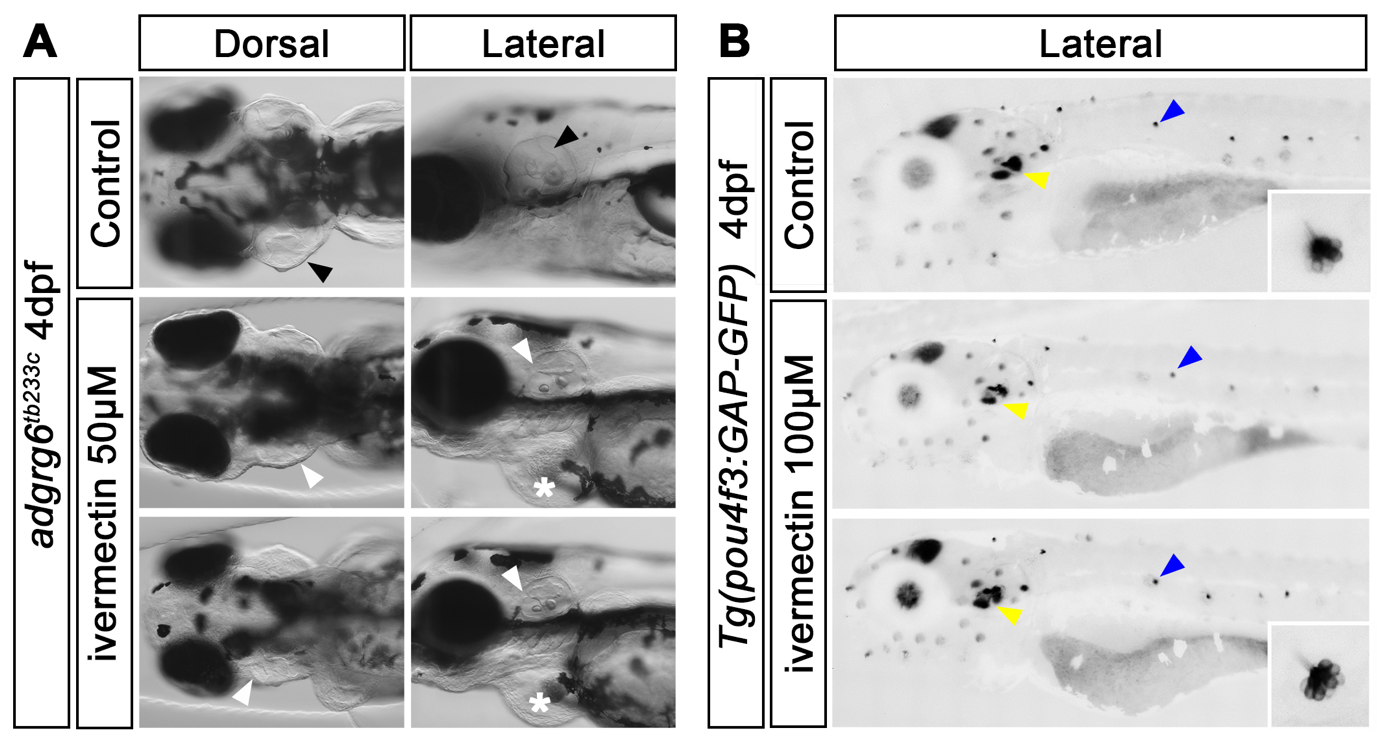


**Figure S5. Performance of ivermectin in zebrafish toxicity assays.** (A) Treatment with 50 µM ivermectin for 44 h gives a partial rescue of ear swelling and fusion of semicircular canal projections in homozygous hypomorphic *adgrg6^tb233c-/-^* mutants. Black arrowheads mark the swollen ear in control (untreated) mutants; white arrowheads mark partial rescue of the ear phenotype in two individual mutant embryos. Treatment at this dose is cardiotoxic; the asterisk marks pericardial oedema, not present in controls. (B) No gross changes in GFP fluorescence (inverted GFP channel) in sensory hair cells of the maculae in the ear (yellow arrowhead) and lateral line neuromasts (blue arrowhead) were observed after treatment with 100 µM ivermectin for 44 h. Insets show enlargements of representative individual posterior lateral line neuromasts, with healthy hair cells visible.

**References**

1. Diamantopoulou E, Baxendale S, de la Vega de León A, et al. Identification of compounds that rescue otic and myelination defects in the zebrafish adgrg6 (gpr126) mutant. *Elife*. 2019;8. doi:10.7554/elife.44889
